# Treatment of tuberculous aortic pseudoaneurysm associated with vertebral tuberculosis: A case series and a literature review

**DOI:** 10.1097/MD.0000000000010382

**Published:** 2018-04-13

**Authors:** Jing Xue, Yimin Yao, Limin Liu

**Affiliations:** a Department of Orthopaedics, West China Hospital, Sichuan University; b Department of Orthopaedics, PLA 452th Hospital, Chengdu, China.

**Keywords:** mycotic aneurysm, tuberculous aneurysm, tuberculous pseudoaneurysm, vertebral tuberculosis

## Abstract

**Rationale:**

Tuberculous aortic pseudoaneurysm associated with vertebral tuberculosis is a rare disease but with very high mortality. We review the literature and find 19 reports with 22 patients. Here we report three cases with vertebral tuberculosis, who also have tuberculous pseudoaneurysm of the aorta. These patients were treated by different methods. We try to analyze the epidemiology, pathogenesis, presentation, and management of this disease to find the best treatment.

**Patient concerns:**

The patients presented with different symptoms such as pain (chest, abdominal or back), fever, blood volume reduction or hemorrhagic shock symptoms. Large mass also could be observed by imaging. In addition to clinical manifestations, enhanced computed tomography or magnetic resonance imaging could also help the diagnosis of this disease.

**Diagnoses:**

Tuberculous aortic pseudoaneurysm associated with vertebral tuberculosis.

**Interventions:**

Three patients were treated with anti-tuberculosis(TB) drugs or combined with different sequences surgical treatment: Case 1 refused to receive pseudoaneurysm surgery and only had anti-TB drug treatment; Case 2 received thoracic spinal surgery first; Case 3 received endovascular stent grafting.

**Outcomes:**

Two patients (case 1 and case 2) who refused to undergo aneurysm surgery died. The last patient (case 3) underwent endovascular repair and antibiotic therapy for tuberculosis, and the postoperative course was uneventful; the patient recovered and survived.

**Lessons:**

Once the diagnosis of tuberculous pseudoaneurysm is confirmed, surgical treatment should be provided immediately combined with anti-tuberculosis drugs. The aim of the treatment is to save lives, prevent relapse, and facilitate the return to normal life, regardless of the size of the pseudoaneurysm. The pseudoaneurysm should be treated first to prevent aneurysm rupture before the vertebral tuberculosis surgery.

## 1 Introduction

The first case of tuberculous aortitis was reported by Weigert in 1882.^[1]^ Osler described a patient with multiple aneurysm secondary to mycotic endocarditis in 1885.^[2]^ As the lesion resembled a fungal growth, he used the term mycotic aneurysm to describe the disease. The first case of tuberculous mycotic aneurysm was reported by Kamen in 1895.^[3]^ Rob and Eastcott^[4]^ reported the first successful reconstruction of tuberculous mycotic aneurysm of the aorta using an Orlon cloth graft in 1955. Because most tuberculous mycotic aneurysms were false aneurysms, the term tuberculous pseudoaneurysm was widely used.^[5,6]^ In this study, we report 3 cases with tuberculous pseudoaneurysm of the aorta associated with vertebral tuberculosis (TB), The 3 cases were treated by different methods and had different results. We try to discuss the epidemiology, pathogenesis, presentation, and management of this disease to find the best treatment for the disease.

## 2 Method

The patients provided informed consents for the publication of their clinical and radiological dataset. This case report was approved by Medical Ethical Committee of West China Hospital, Sichuan University.

Patients with a history of tuberculous pseudoaneurysm of the aorta associated with vertebral TB were included. The patients’ presentation, diagnosis, management, imaging data, angiography findings, and prognosis were collected for analysis.

## 3 Results

Case 1 refused to receive pseudoaneurysm surgery and had only anti-TB treatment. During the follow-up, chest and back pains persisted accompanied by intermittent cough and hemoptysis. He died of sudden massive hemoptysis in the 2nd year after discharge. Case 2 had the thoracic spinal surgery before the pseudoaneurysm surgery. Unfortunately, she died of a sudden rupture of the pseudoaneurysm during the operation. Case 3 had the endovascular stent grafting. After 1 and a half years’ follow-up, her symptoms and signs disappeared without pseudoaneurysm recurrence by the computed tomography (CT) examination.

### 3.1 Case 1

**Figure 1:**
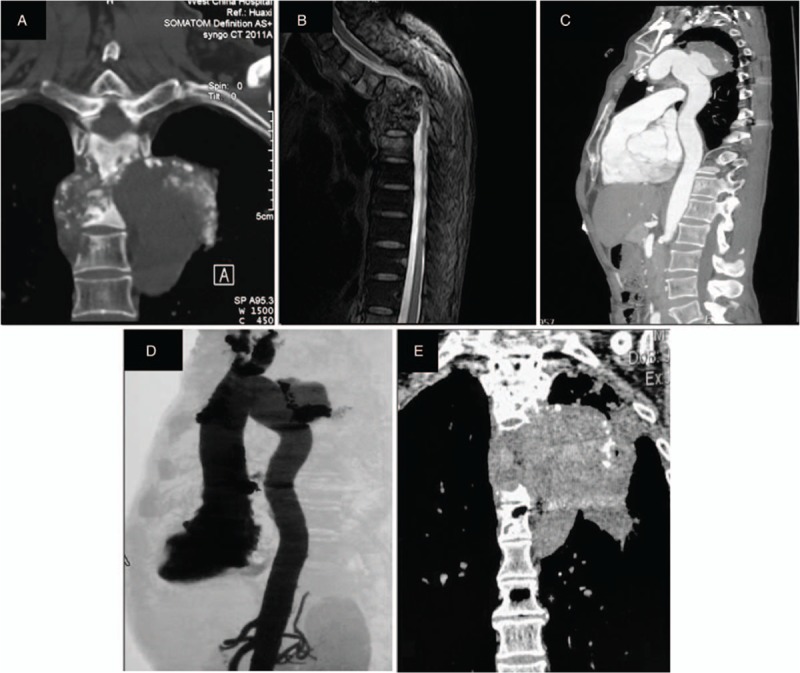
Patient 1: CT (A) and MRI (B) reveal multiple bone destruction of T2–T6 vertebrae with peripheral abscess formation. Enhanced CT (C) and angiography (D) show the descending aorta aneurysm around the T4 and T5 level. CT shows the paravertebral mass has expanded in the end of follow-up (E). CT = computed tomography, MRI = magnetic resonance imaging.

A 55-year-old man was admitted in August 2013 with complaints of recurrent chest and back pain over the past 5 years. Because of symptom aggravation for 6 months, the patient visited the hospital, with persistent distending pain, discomfort, and night sweats but without fever. The laboratory examination showed the following: white blood cell count (WBC) 6.35 × 10^9^ cells/L, erythrocyte sedimentation rate (ESR) 63 mm/hour, C-reactive protein (CRP) 24.5 mg/L, hemoglobin 144 g/L, albumin 39.5 g/L, and HIV-ab negative. Chest CT showed multiple pulmonary nodules; magnetic resonance imaging (MRI) revealed multiple thoracic vertebra bone destruction with peripheral abscess formation, suggesting a TB infection (Fig. 1A, B). The patient was initially diagnosed with T2–6 TB with paravertebral abscess formation. Curettage of T2–6 TB was performed through posterior surgery, and internal fixation with autologous iliac crest bone graft fusion was performed. Intraoperative pus examination revealed a positive result in the acid-fast staining.

After the operation, the patient continued receiving isoniazid (0.3 g/day), rifampin (0.45 g/day), and ethambutol (0.75 g/day) during the follow-up. Nine months postoperatively, the patient presented with recurring chest and back pain, which was intermittently tingling. Repeated chest CT suggested a thickened aortic arch, and the boundary between the paravertebral mass and the aorta was unclear. Enhanced CT and angiography suggested an approximate 2.1-cm breach on the right posterior wall of the upper portion of the descending aorta, which is around the T4 and T5 levels and where the contrast agent entered toward the paravertebral mass, further suggesting the existence of an aortic pseudoaneurysm (Fig. 1C, D). The patient's ESR and CRP were 5 mm/hour and 3.82 mg/L, respectively; his hemoglobin was 133 g/L and albumin was 39.1 g/L. As tuberculous pseudoaneurysm is potentially lethal, the patient was recommended to undergo surgery and receive simultaneous anti-TB treatment. However, the patient refused surgery and only had anti-TB treatment. During the follow-up, his chest and back pains persisted and were accompanied by intermittent cough and hemoptysis. Repeated CT showed the paravertebral mass had expanded than before (Fig. 1E). Unfortunately, the patient died of sudden massive hemoptysis 2 years after discharge.

### 3.2 Case 2

**Figure 2:**
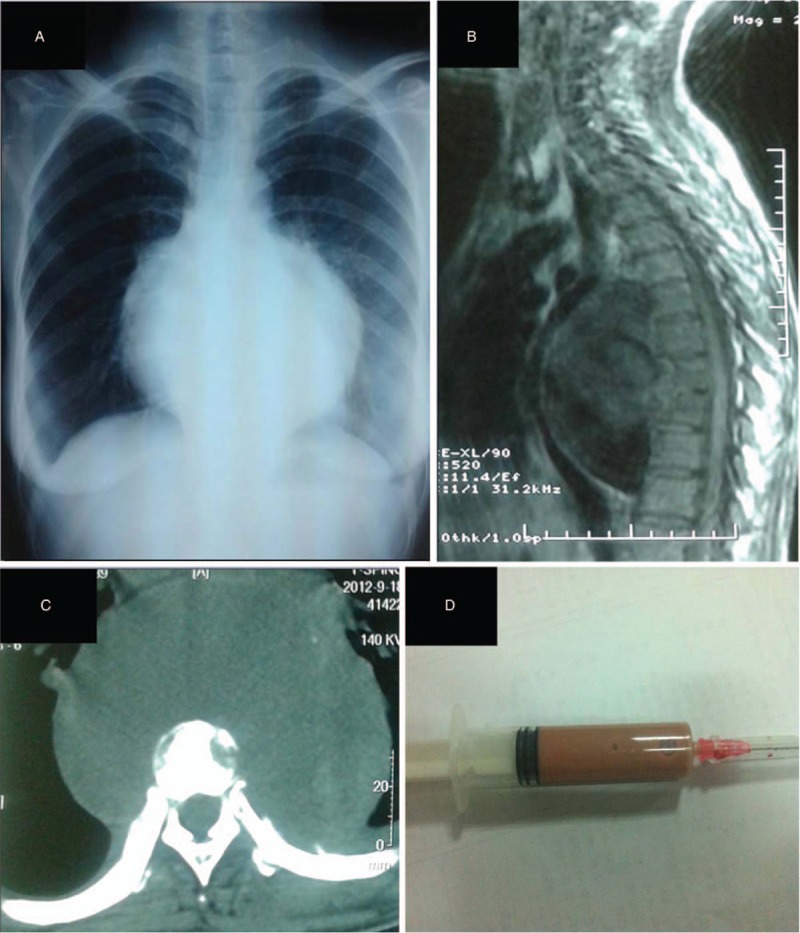
Patient 2: Plain radiography (A), MRI (B), and CT (C) show a paravertebral mass in front of T5–T11 with erosion of the vertebrae. The acid-fast staining of the pus was positive (D). CT = computed tomography, MRI = magnetic resonance imaging.

A 33-year-old woman was admitted for lower back pain for 5 months in September 2012. She had intermittent distending pain at the beginning of her illness, and the pain became persistent with intermittent tingling. No prominent aggravating or alleviating factors were noted, and the pain did not aggravate at night. The patient also presented with fever, night sweats, and significant weight loss. In addition, the patient preferred to lie in a lateral flexion position. Her WBC count was within the normal range, ESR 22 mm/hour, CRP 37.8 mg/L, hemoglobin 89 g/L, albumin 30.4 g/L, and HIV-ab negative. Thoracic vertebral radiography suggested T8–9 bone destruction with paraspinal abscess formation. Chest CT showed scattered nodules in the lung, suggesting pulmonary TBs. CT and MRI of the thoracic spine revealed a large irregular shadow with mixed signal intensities in front of T5–11; the largest diameter was 13.5 cm, pushing the thoracic aorta. In addition, an approximately 1.9-cm tear on the thoracic aorta could be seen connecting to the mass (Fig. 2A–C). The patient was initially diagnosed with thoracic spinal TB, with paraspinal abscess formation and tuberculous pseudoaneurysm of the thoracic aorta. Because of severe chest and back pains, the patient was unable to sit and walk; thus, thoracic surgery was necessary to restore the stability of the spine first. The surgery for tuberculous aneurysm of the thoracic aorta was temporarily postponed. However, a sudden rupture of the pseudoaneurysm occurred during the operation, and the patient died after an unsuccessful emergency treatment. During the operation, a 10 × 15 × 10-cm mass was observed in front of the spine. It adhered to the left lung and was closely attached to the thoracic aorta and thus difficult to separate. Moreover, a large amount of blood clots was visible in the mass. Caseous necrotic tissues and pus (Fig. 2D) were removed during the operation, and the acid-fast staining was positive.

### 3.3 Case 3

**Figure 3:**
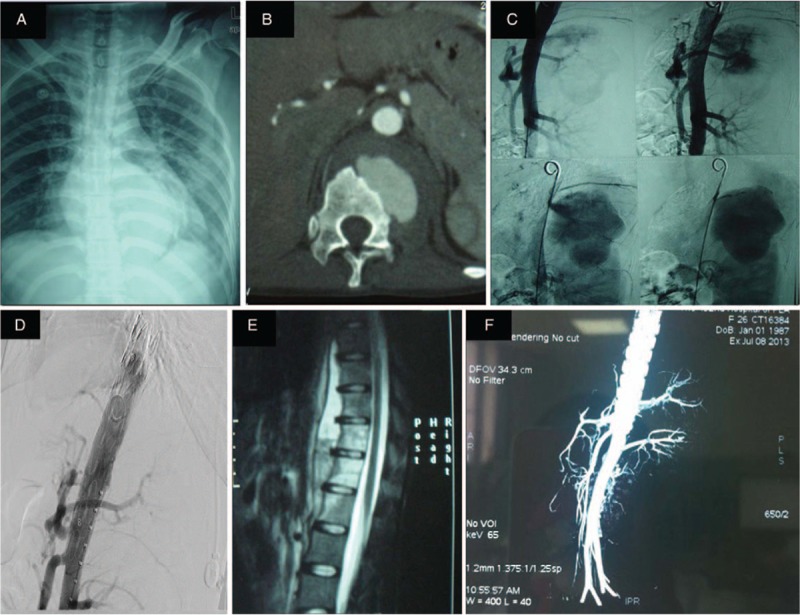
Patient 3: Plain radiography (A) showed T6–T10 bone destruction with paraspinal abscess formation. Enhanced computed tomography (CT) (B) and angiography (C) show the pseudoaneurysm on descending aorta. Endovascular stent grafting (D) was performed to treat the aneurysm, and repeated angiography confirmed a complete closure of the mass. The patient had good stability of the spine (E). After 18 months's follow-up, repeated CT (F) showed no pseudoaneurysm recurrence.

A 24-year-old woman was admitted for low back pain for 3 months in December 2011. The pain was persistent with no significant aggravating or mitigating factors and no aggravation at night. The patient presented with no hectic fever and night sweats, but had significant weight loss. The patient's WBC count was within the normal range, and other laboratory exams showed the following: ESR 25 mm/hour, CRP 37.4 mg/L, hemoglobin105 g/L, albumin 30.4 g/L, and HIV-ab negative. Thoracic spinal radiography showed T6–10 bone destruction with paraspinal abscess formation. Chest CT showed scattered grain-like nodules in both lungs, suggesting TB, and bone destruction in the right anterior vertebrae; the boundary between the paraspinal abscess and thoracic aorta was unclear (Fig. 3A). Enhanced CT suggested an approximately 1-cm tear on the thoracic aorta connected to the paraspinal abscess where the contrast agent entered toward the paraspinal abscess (3.5 × 2.5 cm) (Fig. 3B). Angiography showed that a large amount of contrast agent entered into the paravertebral mass through the rupture on the thoracic aorta (Fig. 3C). Endovascular stent grafting was performed to treat the aneurysm, and repeated angiography confirmed a complete closure of the mass, a smooth blood flow in the aorta, and a firmly fixed stent on the aortic wall without distortion and displacement; no peripheral leakage of the contrast agent was observed (Fig. 3D). The operation was successful, and the patients continued receiving anti-TB drugs after the operation. The patient had good stability of the spine; thus, spinal stability reconstruction was not considered (Fig. 3E). After 18 months's follow-up, the patient's symptoms and signs disappeared, and repeated CT showed no pseudoaneurysm recurrence (Fig. 3F).

## 4 Discussion

### 4.1 Epidemiology

Tuberculous pseudoaneurysm is a rare disease, and the case with spinal TB is even rarer. Between 1902 and 1951, 338 of the 22,792 autopsies in Boston City Hospital had aortic aneurysms, and only 1 case was tuberculous aneurysm.^[7]^ Of the 96 patients with tuberculous pseudoaneurysm reported by Jain et al,^[8]^ only 6 patients had spinal TB. We reviewed previous reports and found that only 19 related reports exist and only 22 patients with tuberculous pseudoaneurysm had spinal TB.^[8–26]^ (Table 1) With our 3 cases, a total of 25 cases are reported.

**Table 1:**
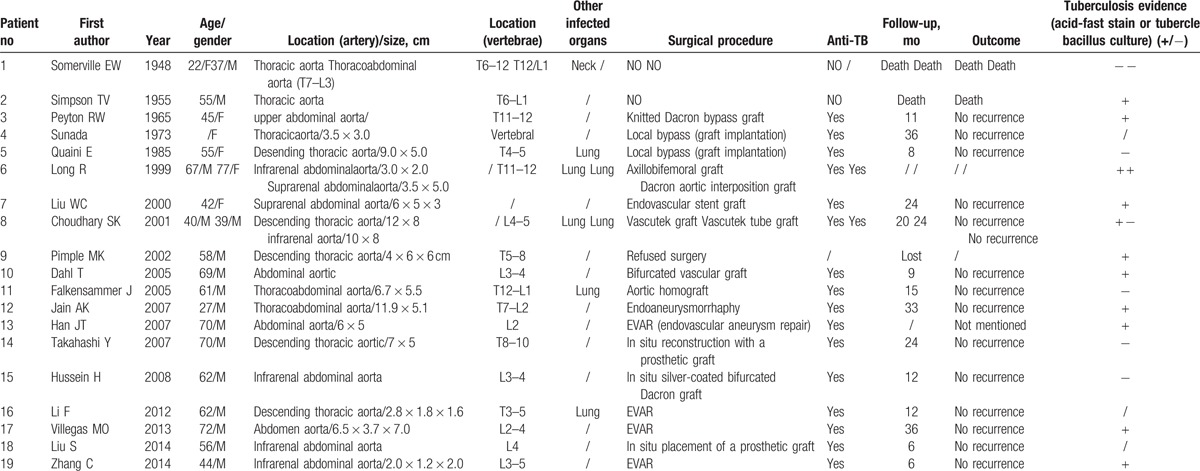
Review of literature about tuberculous pseudoaneurysm associated with vertebral tuberculosis.

### 4.2 Pathology and pathogenesis

In the study by Long et al,^[14]^ the pseudoaneurysms in 19 patients were located in the thoracic aorta, 21 in the abdominal aorta, and only 1 at the junction of the thoracic and abdominal aorta. Of the 15 new cases added by Forbes et al,^[27]^ 9 were located in the thoracic aorta, 5 in the abdominal aorta, and 1 at the junction of thoracic and abdominal aorta. We found tuberculous pseudoaneurysms located in the thoracic aorta, abdominal aorta, and junction of thoracic and abdominal aorta in 8, 11, and 3 cases, respectively; the 3 cases in our study were all located in the thoracic aorta. Thus, TB pseudoaneurysm often occurs in the thoracic and abdominal aorta. Moreover, the pseudoaneurysms are often cystic-like rather than spindle-like,^[14,26]^ and tuberculous pseudoaneurysm may form through the following mechanisms^[28]^: *Mycobacterium tuberculosis* reaching the vessel wall through feeding vessels; spread of bacteria through lymphatic vessels around the artery; direct inoculation of the *M tuberculosis* after vasculature trauma; and (4) direct invasion and spread from lymph nodes, abscess, and bone TB around the artery. Of these 4 mechanisms, the last is the most common mode of transmission, accounting for approximately 75% of cases.^[14]^ After direct damage to the full thickness of the vessel wall due to peripheral lymphadenitis, pericarditis, or paravertebral abscess, hemorrhage and perivascular hematoma formation occur, fibrosis gradually forms in the periphery of hematoma, and the hematoma is enwrapped. The rupture is connected to the lumen of the aorta; subsequently, the so-called pseudoaneurysm is formed, which is why most of tuberculous aortic aneurysms are pseudoaneurysm.^[29]^ All 3 patients presented here had tuberculous damage around the aneurysm, which was accompanied by abscess formation. Pathological examination showed caseous necrosis, and examination for *M tuberculosis* was positive. Therefore, in all 3 patients, the pseudoaneurysms were formed through the 4th transmission mechanism. Furthermore, tuberculous aortic aneurysms at the junction of the thoracoabdominal aorta are rare possibly because of the few surrounding lymph nodes and soft tissues in this region.^[30]^

### 4.3 Presentation

Among the 25 cases of tuberculous aortic aneurysm with spinal TB, 10 cases had pulmonary TB, which accounted for approximately 40% of all cases and indicated that a high proportion of patients with tuberculous aortic aneurysm simultaneously had disseminated TB. Majority of the patients presented with more than one clinical presentation. Majority of the patients presented with more than one clinical presentation:

(1) Persistent chest pain, abdominal pain, or back pain (21 cases: 9 with thoracic aortic aneurysm, 10 with abdominal aortic aneurysm, and 2 with aneurysm at the thoracoabdominal junction).
(2) Fever (10 cases: 4 with thoracic aortic aneurysm, 4 with abdominal aortic aneurysm, and 2 with aneurysm at the thoracoabdominal junction).
(3) Blood volume reduction (blood flows into the interlayer of the aneurysm, which in turn leads to reduced circulating blood volume) or hemorrhagic shock symptoms (blood enters into the thoracic cavity, retroperitoneal space, and pericardium) (3 cases: 1 with abdominal aortic aneurysm and 2 with thoracic aortic aneurysm).
(4) Large mass that is clearly visible, can be observed by imaging, or is pulsatile or gradually enlarges (25 cases: 11 with thoracic aortic aneurysm, 11 with abdominal aortic aneurysm, and 3 with aneurysm at the thoracoabdominal junction).

Pain and the presence of mass are the most common. Other symptoms include night sweats, cough, weight loss, and hemoptysis. Therefore, tuberculous aortic pesudoaneurysm should be considered in patients with TB presenting with the above clinical features, especially with the symptoms of pain or paraaortic mass.^[15]^

Previously, with an underdeveloped imaging technology, tuberculous pseudoaneurysms are typically identified by autopsy.^[9,10]^ With increased health consciousness and the advancement of imaging technology, the diagnosis of the disease has improved. In general, arterial radiography (X-ray) has little significance; nevertheless, for tuberculous pseudoaneurysm with spinal TB, radiography can show vertebral or intervertebral space damage, which could aid in the diagnosis of vertebral or intervertebral space infection. Enhanced CT exam can clearly show images of the surrounding blood vessels, including lymph nodes and abscesses; thus, it has gradually been used to assist in the diagnosis of aneurysms. Moreover, since its first application in the diagnosis of aneurysm by Harris and Hougen in 1978,^[31]^ contrast-enhanced CT scan has become an effective approach for the early diagnosis of aneurysm.^[29]^ In our study, the paravertebral mass in almost all cases was identified by imaging exams. In addition to radiography and enhanced CT, MRI can also be employed.^[14,17,29]^

Simpson and Grobbelaar^[10]^ showed that positive acid-fast staining or *M tuberculosis* culture was noted in 8.6% (3/35) only. In our study, of 25 cases, 15 (60%) showed positive indicators. Therefore, negative acid-fast staining or *M. tuberculosis* culture could not be used as an exclusion criterion. When patients present with fever, cough, night sweats, weight loss, and hemoptysis, accompanied by chest and back pain or palpable or imaging-visible paravertebral mass, especially in the case of pulmonary TB and in those with a gradually enlarged mass in the follow-up imaging exams, the existence of the disease should be highly suspected. Positive results in the acid-fast staining or culture further confirm the possibility of tuberculous pseudoaneurysm.

### 4.4 Management

Tuberculous aneurysm was first reported by Kamen in 1895.^[3]^ The first attempted surgical repair was performed by Herndon in 1949.^[32]^ However, the patient died on the 6th postoperative day. Only until 1955, after Rob and Eastcott used Orlon cloth graft to reconstruct the aorta damaged by the aortic aneurysm, that cases of this disease were successfully treated by surgery.^[4]^ Currently, surgery combined with simultaneous anti-TB drug treatment should be used for the disease,^[26,27]^ especially considering that no evidence showing that only anti-TB drug treatment or surgery alone can achieve a cure exists.^[6,14]^ Moreover, as tuberculous pseudoaneurysm is mainly caused by inflammatory reactions surrounding the arteries, revascularization and fibrosis may lead to adhesion between blood vessels and their surrounding tissues. Consequently, surgical treatment becomes challenging because of the difficulty of separating blood vessels and the surrounding tissues, which also increases the risk of aneurysmal rupture.^[24]^ Hence, once a patient is suspected of having the disease, initial treatment with anti-TB drugs is recommended for the improvement of the arterial wall and the surrounding soft tissues, thereby reducing the incidence of postoperative complications and mortality rate.^[17]^ Of note, surgical treatment should be immediately performed once the diagnosis of tuberculous pseudoaneurysms is confirmed, regardless of the tumor size; even a 1-cm aneurysm has the possibility of rupture.^[33]^ Because the effect of anti-TB drugs on the aneurysm wall and *M tuberculosis* is limited,^[34]^ and the aneurysm may suddenly enlarge and even rupture, causing death of patients during the course of treatment,^[14]^ surgery should not be delayed once the diagnosis of tuberculous pseudoaneurysm is confirmed.

Common surgical methods include the following: vascular lesion removal and synthetic vascular replacement; extra-anatomic reconstruction; direct suture closure or patch repair; and endovascular stent-graft exclusion or endovascular aneurysm repair (EVAR). Currently, the most common surgical approach involves the resection of the diseased segment, removal of the surrounding necrotic tissues, and reconstruction of the distal vessel using a graft from the noninfectious region far from the infected areas (anatomic bypass).^[26]^ However, because of large surgical trauma, such approach is not suitable for elderly patients with poor physical condition and who cannot tolerate open surgery. In addition, since 1990s, EVAR has been used to treat tuberculous pseudoaneurysm because it is less invasive and offers good results.^[23,24,26]^ However, EVAR also has drawbacks, such as transplant site infection, later-stage prosthesis rupture, and embolism.^[15]^

Furthermore, we believe that the choice of surgical approaches should depend on the patients’ specific conditions. For example, if pleural or intraabdominal conditions permit and the lesion segment is not long, direct resection of the aortic lesion and synthetic vascular reconstruction should be used. In case of severe infection or infection of a large segment of the vessel, extra-anatomic bypass reconstruction can be used. In elderly patients with a poor general condition and who cannot tolerate surgery and in cases of suddenly enlarged aneurysm, which is on the brink of rupture, EVAR is recommended to avoid life-threatening pseudoaneurysm rupture. Moreover, EVAR also provides patients time to correct poor general condition and attain recovery. Further surgery to remove lesions can be performed thereafter.

In our study, because of vertebral destruction and spinal instability, vertebral surgery was also needed at a later stage. Nevertheless, aortic pseudoaneurysm treatment must be provided first to reduce the possibility of pseudoaneurysm rupture; subsequently, surgery for spinal TB to avoid life-threatening pseudoaneurysm rupture should be performed.

## 5 Conclusions

In conclusion, given that tuberculous pseudoaneurysm has severe and fatal complications, once the disease is suspected, examinations should be immediately conducted to confirm the diagnosis, especially for patients with pulmonary TB presenting with persistent chest and abdominal pain and thoracoabdominal paraspinal pulsatile mass. Moreover, a patient would likely be diagnosed with tuberculous pseudoaneurysm if enhanced CT, MRI, or aortic angiography shows a large contrast agent-filled mass around major arteries. Once the diagnosis of tuberculous pseudoaneurysm is confirmed, surgical treatment should be provided immediately combined with anti-TB drugs to save lives, prevent relapse, and facilitate the return to normal life, regardless of the size of the pseudoaneurysm. The pseudoaneurysm should be treated first to prevent aneurysm rupture before the vertebral TB surgery.

## Author contributions

**Conceptualization:** Jing Xue.

**Data curation:** Jing Xue.

**Formal analysis:** Jing Xue.

**Investigation:** Jing Xue.

**Methodology:** Limin Liu.

**Project administration:** Limin Liu.

**Resources:** Yimin Yao.

**Software:** Limin Liu.

**Writing – original draft:** Jing Xue.
